# Determinants for the Development of Visceral Leishmaniasis Disease

**DOI:** 10.1371/journal.ppat.1003053

**Published:** 2013-01-03

**Authors:** Laura-Isobel McCall, Wen-Wei Zhang, Greg Matlashewski

**Affiliations:** Department of Microbiology and Immunology, McGill University, Montreal, Quebec, Canada; International Centre for Genetic Engineering and Biotechnology, India

## Abstract

Leishmaniasis is a vector-borne neglected tropical disease associated with a spectrum of clinical manifestations, ranging from self-healing cutaneous lesions to fatal visceral infections. Among the most important questions in *Leishmania* research is why some species like *L. donovani* infect visceral organs, whereas other species like *L. major* remain in the skin. The determinants of visceral leishmaniasis are still poorly understood, although genomic, immunologic, and animal models are beginning to provide important insight into this disease. In this review, we discuss the vector, host, and pathogen factors that mediate the development of visceral leishmaniasis. We examine the progression of the parasite from the initial site of sand fly bite to the visceral organs and its ability to survive there. The identification of visceral disease determinants is required to understand disease evolution, to understand visceral organ survival mechanisms, and potentially to develop better interventions for this largely neglected disease.

## Introduction

Leishmaniasis is a neglected tropical disease caused by *Leishmania* protozoa that are transmitted to mammalian hosts by an infected sand fly. *Leishmania* have a dimorphic life cycle in which parasites reside as extracellular promastigotes in the vector and as intracellular amastigotes in mammalian host macrophages [1]. Leishmaniasis includes a spectrum of clinical manifestations, from cutaneous lesions at the site of sand fly bite to systemic visceral leishmaniasis (Table 1). The distinct clinical manifestations are associated with different degrees of parasite metastasis, from parasites such as *L. major* that remain contained at cutaneous lesions to dissemination into the visceral organs in visceral leishmaniasis caused by *L. donovani*. We focus here on the most severe form of the disease, visceral leishmaniasis.

**Table 1:** *Leishmania* species and disease phenotype.

Visceral leishmaniasis	Common	*L. donovani*
		*L. infantum*
		*L. chagasi* ( = *L. infantum*)
	Rare	*L. tropica*
		*L. amazonensis*
Cutaneous leishmaniasis	Common	*L. major*
		*L. tropica*
		*L. amazonensis*
		*L. mexicana*
		*L. braziliensis*
	Rare	*L. infantum*
		*L. donovani*
Mucocutaneous leishmaniasis	Common	*L. braziliensis*
	Rare	*L. panamensis*
		*L. guyanensis*
		*L. amazonensis*

Visceral leishmaniasis is ranked second in mortality and fourth in morbidity among tropical diseases, with 20,000 to 40,000 deaths per year [2] and over 2 million DALYs (disability-adjusted life years) lost [3]. Symptoms include hepatosplenomegaly, high fever, pancytopenia, and hypergammaglobulinemia, and the disease is almost always fatal if untreated [1], [4]. Identification of the factors that mediate the development of visceral leishmaniasis is highly relevant to understanding disease progression and parasite survival mechanisms.

## Navigating from Skin to Viscera

The sand fly vector lacerates blood vessels during feeding, so parasites are introduced intradermally into a pool of blood [5]. Free amastigotes have been detected in the bloodstream and could be directly delivered to blood-filtering organs [6]. Alternatively, the eventual spread to visceral organs could involve the movement of infected cells. Neutrophils are the earliest cells recruited to the site of the sand fly bite [7], [8] and represent the first infected cell population [9]. Infected neutrophils or free parasites are then taken up by dendritic cells and macrophages, which migrate away from the site of the bite [9]. The dermal dendritic cell population infected at early time points may differ between *L. major* and *L. donovani* [7], and there is evidence that *L. donovani*–infected macrophages and dendritic cells leave intradermal injection sites in higher numbers than *L. major*–infected cells [10]. However, the route used by infected cells to eventually reach the visceral organs remains poorly understood.

Experimental subcutaneous needle infection with *L. major* in BALB/c mice is associated with parasite proliferation and lesion development at the site of injection, as well as parasite dissemination to visceral organs [11]. Dissemination of *L. major* to the visceral organs does not occur in C57BL/6 mice, indicating an important role for the host immune response in the control of visceralization [11]. In comparison, subcutaneous injection of *L. donovani* in BALB/c mice only causes minimal swelling at the site of injection and no dissemination to the viscera [12]. Therefore, these subcutaneous infection models do not accurately reflect the situation in humans where *L. donovani*, but not *L. major*, metastasizes to the visceral organs.

Intradermal infection models have been developed in BALB/c mice [13] and in hamsters [14] in which parasites are cleared from the skin and disseminate to the visceral organs. However, for both models, the inoculation dose is 100- to 1,000-fold higher than the natural sand fly inoculum [15]. Nevertheless, these models more closely mimic natural infection routes for visceral disease and could provide better insight into the cells and pathways involved in reaching the visceral organs.

## Surviving Stress: Parasite Proliferation in the Viscera

Experimental intravenous injection of *Leishmania* in mice bypasses the normal requirement for the parasite to transit from skin to viscera and therefore focuses exclusively on parasite survival within the liver and spleen. However, although intravenous infection models of BALB/c mice are commonly used to study visceral leishmaniasis, these do not fully reflect human visceral leishmaniasis progression. In mice, infection in the liver is self-resolving, while spleen infection is progressive, and overall infection is asymptomatic [16]. In contrast, infection of hamsters is associated with symptomatic disease and can be fatal [17].

Nevertheless, intravenous infection with cutaneous species such as *L. major* is associated with limited liver and spleen parasite burden, while intravenous infection with viscerotropic *L. donovani* and *L. infantum* results in high levels of visceral infection [18]. Therefore, regardless of the mechanism to exit the skin, cutaneous and visceral species differ dramatically: visceral species are much better adapted to survive and proliferate in visceral organs than cutaneous species.

Temperatures within infected mouse footpad dermis vary between 28°C and 32°C [19], whereas fever in visceral leishmaniasis exceeds 40°C [20]. Viscerotropic parasites must therefore withstand higher temperatures than cutaneous ones, and indeed, promastigotes from cutaneous species are considerably more sensitive to heat shock than promastigotes of visceral species [21], [22]. In addition, transfection of some *L. donovani* genes into *L. major* enhanced *L. major* survival at higher temperatures and visceralization [18], [21], [23].

Fever itself can also augment the immune response by increasing dendritic cell and neutrophil migration, pro-inflammatory cytokine production, and Th1 cell activity [24]. Given that fever increases oxidant production by phagocytic cells [24], viscerotropic species are expected to be more resistant to oxidants than cutaneous species: *L. donovani* is more resistant to nitric oxide (NO) and hydrogen peroxide than *L. major* [25].

The host macrophage population targeted by *Leishmania* also differs between cutaneous and visceral species: cutaneous species infect inflammatory monocyte-derived macrophages and dendritic cells [9], [26], while visceral species infect Kupffer cells, spleen macrophages, and bone marrow macrophages [27]. These different macrophage populations express different levels of cell surface molecules [28] and of NRAMP1, a cation transporter associated with resistance to *Leishmania* [29]. They also differ in their response to IFNγ stimulation, and in their capacity to produce cytokines, activate T lymphocytes, and kill pathogens [30]–[32]. Therefore, cutaneous and visceral species have adapted to replicate in distinct host macrophage environments. However, no direct comparisons of the susceptibility and killing potential of these different macrophage populations have been performed during *Leishmania* infection.

## Host Determinants of Visceral Leishmaniasis

The ratio of subclinical to symptomatic visceral leishmaniasis is estimated at up to 18 to 1 [33], demonstrating that many people infected with visceral *Leishmania* species develop an effective immune response and do not manifest clinical disease. The host genetic background influences the development of disease [34], [35] (reviewed in [36]–[38]). In particular, NRAMP1 plays a key role in susceptibility to visceral disease [39]. A number of cytokines, chemokines, and their receptors (TNFα [40]; IL4 [41]; TGFβ [42]; IL2 receptor [43]; CXCR2 [44]), as well as mannan-binding lectin [45] and the Delta-like 1 ligand for Notch 3 (DLL1) [46], have also been associated with symptomatic *versus* asymptomatic disease. However, it is largely unknown how deeply polymorphisms in these host immune response genes penetrate to cause visceral leishmaniasis in the human population of endemic regions.

In addition to genetic factors, acquired characteristics, such as AIDS (reviewed in [47]), preexposure to the parasite in utero [48], malnutrition [49], [50], and youth [51], [52], can also increase the risk of developing symptomatic leishmaniasis. All of these are associated with impaired immune responses against the parasite. Visceral leishmaniasis in HIV-coinfected individuals can be caused by strains and species that normally cause cutaneous disease [53] and is associated with atypical disease manifestations, such as the presence of cutaneous lesions as well as visceral parasitemia [54].

Overall, these observations indicate that an effective Th1 cellular immune response is required to control infection. This response involves the release of IL12 by antigen-presenting cells, leading to the differentiation of Th1 cells. These release IFNγ, resulting in macrophage activation and the production of leishmanicidal NO [55]. Decreased NO production promotes visceral dissemination following cutaneous infection with *L. major* in mice [56]. Conversely, subcutaneous self-limiting infection by *L. donovani* has been associated with strong NO production [12]. However, the Th1/IFNγ-mediated response alone is not sufficient to protect against disease, since visceral leishmaniasis patients are able to produce IFNγ in response to *Leishmania* antigen [57]. Disease is strongly correlated with the production of high amounts of IL10 [57], [58], an immunosuppressive cytokine that inhibits leishmanicidal immune functions (reviewed in [59]).

## Vector Determinants of Visceral Leishmaniasis

*L. chagasi* transmitted by *Lutzomyia longipalpis* sand flies can cause cutaneous or visceral leishmaniasis in parts of South America. Saliva of sand flies from a cutaneous region caused low levels of vasodilatation and promoted footpad swelling following subcutaneous infection in mice, whereas saliva of sand flies collected in a visceral region caused higher vasodilatation but did not enhance cutaneous lesion formation [60]. This suggested that higher vasodilatation promoted better parasite access to the visceral organs and that vector species may influence disease development. However, this may not be a general difference between vectors of cutaneous and visceral *Leishmania* species: sequence analysis of salivary gland proteins from a *Phlebotomus* species associated with cutaneous *L. infantum* cases in the Old World showed that its salivary proteins were more closely related to those of vectors that transmit visceral *L. donovani* and *L. infantum* than to vectors of cutaneous *L. major* and *L. tropica* [61].

The number of parasites transmitted by sand flies could also influence disease outcome. A higher infective dose may promote a stronger local immune response that limits the parasite spread, thereby preventing dissemination to the visceral organs [15].

Finally, immunity to sand fly salivary proteins has been associated with protection against visceral leishmaniasis in hamsters [14] and may also be protective in humans [62]–[64]. The role of the sand fly in disease pathology is an area that requires more attention.

## Parasite Determinants of Visceral Leishmaniasis

Although vector and host characteristics influence the progression to symptomatic disease, parasite characteristics are the most important determinant that distinguishes cutaneous from visceral disease. For example, although *L. infantum* has been associated predominately with visceral leishmaniasis, some subspecies can also cause cutaneous leishmaniasis [65]. Subspecies-specific *L. infantum* differences were maintained following intravenous infection of inbred mice: *L. infantum* strains from cutaneous patients were unable to establish visceral infection, unlike *L. infantum* strains from visceral patients [66]. This demonstrated that genetic differences between *L. infantum* subspecies determine disease pathology. Differences in species-specific genes, gene polymorphisms, pseudogenes, and expression levels for virulence and stress response genes can all contribute to differences in disease pathology.

The A2 gene family represents the prototype example of a gene required for visceralization. It is expressed in *L. donovani* and *L. infantum*, whereas it is a pseudogene in some cutaneous species such as *L. major* and *L. tropica* [67], [68]. A2 genes are arranged in tandem arrays on chromosome 22 [69] and encode a family of proteins from 42 to 100 kDa that are made up almost entirely of 40 to 90 copies of the same repetitive ten amino acid sequence [70]. Downregulation of A2 by antisense RNA [71] or partial knockout of A2 genes [18] resulted in decreased liver parasite burden in BALB/c mouse infection. Conversely, introducing A2 genes into *L. major* enhanced the ability of *L. major*–infected cells to migrate out of the dermis and increased parasite survival in visceral organs [10]. Likewise, expression of *L. donovani* A2 in *L. tarentolae* (a lizard *Leishmania* species) enhanced *L. tarentolae* survival in mouse visceral organs [72]. Finally, A2 expression is downregulated in human post kala-azar dermal leishmaniasis (PKDL) where the *L. donovani* parasite relocates to the skin following successful treatment [73]. Overall, these results indicate a key role for A2 in parasite survival in the visceral organs. A2 gene expression is induced by promastigote to amastigote differentiation [74] and by a variety of stresses, including heat shock [21], unfolded protein stress (UPR) [75], and misfolded protein stress [76]. A2 protects from heat shock [21] and oxidative stress [77], and this may allow the parasite to survive in the warmer visceral organs as well as withstand host defences. It is noteworthy that A2 is now a leading candidate for a visceral leishmaniasis vaccine in dogs and potentially humans [78].

Completion of the *L. major, L. donovani, L. braziliensis,* and *L. mexicana* genomes [79] has highlighted only 19 *L. donovani*–specific genes (out of over 8,000 genes) that are absent or found as pseudogenes in cutaneous species [80]. The ability of some of these genes to promote parasite survival in visceral organs has been investigated by ectopically expressing them in *L. major* and monitoring spleen and liver parasite burden in BALB/c mice [81]–[83]. Interestingly, three *L. donovani*–specific genes could promote *L. major* survival in the viscera, including the orthologues of *LinJ.28.0340, LinJ.15.0900,* and *LinJ.36.2480* [82]. A list of genes identified in this way as potentially involved in visceral disease is presented in Table 2. Knocking out *LinJ.28.0340* in *L. donovani* also decreased parasite survival in the visceral organs [82]. *LinJ.28.0340* is a cytosolic protein of unknown function [82], *LinJ.15.0900* is a nucleotide sugar transporter localized in the Golgi apparatus [83], and *LinJ.36.2480* is a cytosolic glyceraldehyde-3-phosphate dehydrogenase, a rate limiting enzyme involved in glucose metabolism and ATP production [82]. The impact of *LinJ.36.2480* on parasite survival in the visceral organs suggests that energy production may be an important difference between cutaneous and visceral species [82]. However, apart from *LinJ.28.0340*, these genes are present in *L. mexicana* [82], [83], and several of them also promote increased footpad swelling, making them general virulence factors, rather than visceralization-promoting factors.

**Table 2:** *Leishmania* genes implicated in the development of visceral disease.

*L. infantum*	*L. major*	*L. mexicana*	*L. braziliensis*	Name and function	Localization	Effect of introduction into *L. major*		Effect of knockout in *L. donovani*	Ref.
						Visceral parasite burden	Footpad parasite burden		
*LinJ.15.0900*	pseudogene	*LmxM.15.0840*	absent	nucleotide sugar transporter	Golgi	Liver: 18× increase	increased footpad swelling	No change	[83]
	*LmjF.15.0840*					Spleen: 20× increase			
*LinJ.22.0670*	pseudogene	*LmxM.22.0691*	absent	A2	Endoplasmic reticulum	Spleen: 3× increase	6–8× decrease	Partial knockout: 2–3× decrease in LDU	[10]
		*LmxM.22.0692*		Stress response				Antisense: 10–25× decrease in LDU	[18]
									[21]
									[71]
									[77]
*LinJ.28.0340*	pseudogene	pseudogene	pseudogene	Hypothetical protein	Cytosol	Liver: 14× increase	2× increase in footpad swelling	Liver and spleen: 200× decrease	[82]
	*LmjF.28.0420*	*LmxM.28.0420*	*LbrM.28.0430*			Spleen: 11× increase			

Although transfection of these *L. donovani*–specific genes into *L. major* increased parasite survival in the visceral organs, none of them were able to fully restore *L. major* virulence to the same level as *L. donovani.* This argues that combinations of *L. donovani*–specific genes and other factors such as gene amplifications, polymorphisms, and differences in posttranscriptional regulation could all play important roles in visceral infection [82].

## Additional Genetic Determinants of Visceral Disease

There are a large amount of chromosome copy number variations between *L. donovani* strains and between *Leishmania* species [79], [84]. Gene dosage effects may alter protein expression levels between cutaneous and visceral species, some of which could influence parasite tropism and pathology. Microarray studies also showed significant differences in mRNA profile between *L. major* and *L. infantum* for proteases, kinases, antioxidants, enzymes involved in carbohydrate and lipid metabolism [85], and surface proteins such as gp46 and proteophosphoglycan family members [86]. However, protein expression in *Leishmania* is largely regulated posttranscriptionally, and only a weak correlation exists between mRNA and protein levels [87].

It is difficult to draw conclusions from proteomic comparison of *L. major* and *L. donovani* due to high variability between species [88]. However, proteomic analysis may be more informative if these techniques are applied to strains of the same species that cause different disease manifestations, such as for example distinct *L. infantum* isolates causing cutaneous and visceral disease [65]. Finally, changes in posttranslational modifications may also be important. Indeed, a different phosphorylation profile was observed for virulent and avirulent *L. donovani* strains during heat shock [89]. Application of these and other proteomic techniques to carefully chosen *Leishmania* isolates may therefore be more effective in identifying relevant determinants of visceral disease.

It is also of interest to consider how genetically distinct *Leishmania* species alter host macrophage gene expression. *L. donovani* or *L. major* induced remarkably similar macrophage gene expression profiles, although *L. donovani* induced higher levels of Cox2 and prostaglandin E synthase than *L. major* [90]. Both of these enzymes are part of the PGE2 biosynthetic pathway, which has been associated with increased visceral organ infection levels [91]. Spermine/spermidine N1-acyl transferase 1, a rate-limiting enzyme of polyamine metabolism, was also higher in *L. donovani*–infected cells [90], and this may increase polyamine production to promote higher levels of parasite growth [92].

*L. major* also induced higher leukocyte recruitment than *L. donovani* in an air pouch model of infection, with increased chemokine, chemokine receptor, and pro-inflammatory cytokine expression [93]. Dendritic cell infection with *L. major* was also associated with higher IL12 production than *L. donovani* infections, which would promote increased T helper cell activation and parasite killing [94]. Similarly, TNFα production by infected monocytes is higher following infection with *L. major* than with *L. infantum* [95]. The higher pro-inflammatory response to *L. major* may restrict it to cutaneous sites and decrease its spread to visceral organs. These and other differential effects on the host macrophage need to be further explored to determine whether they influence the final outcome of infection.

## Perspectives for Future Work

Our understanding of the determinants of visceral disease and the interplay of host, vector, and parasite factors has progressed significantly, and this has led to the conclusion that the genetic differences between species are the main determinant for cutaneous or visceral disease. There are however a number of questions to answer to clarify the evolution of visceral disease and the key parasite genetic differences, and these are outlined in Table 3.

**Table 3:** Unresolved questions.

Mechanism of visceralization
○ Free parasites or infected cells?
○ Via blood or lymph?
○ Similar mechanism for spleen, liver, and bone marrow?
○ Chronology
○ Pathway
○ Natural bite (sand fly model) for visceral species
Identification of new virulence factors required for visceral disease
○ Role of species-specific genes
○ Genomic and proteomic comparison of strains that cause atypical disease phenotypes
○ Generation and comparison of hybrids of cutaneous and visceral species
○ Factors that allow *L. donovani* to switch from a visceral parasite (visceral leishmaniasis) to a cutaneous parasite (PKDL)
Evolution: is visceralization a newly acquired characteristic?
Influence of the reservoir

One of the most promising new approaches to identify new determinants of visceral disease involves sexual crossing of different *Leishmania* species in infected sand flies [96], [97]. This approach could identify key regions of the *L. donovani* genome required for visceral disease. Another promising approach is to closely examine the genome of rare *L. donovani* isolates that cause cutaneous disease, such as for example in Sri Lanka, where *L. donovani* is responsible for an epidemic of cutaneous leishmaniasis [98]. It will be important to determine how these *L. donovani* strains behave in animal models and, through genome sequencing, to identify potential deletions, pseudogenes, and polymorphisms. It may be possible to experimentally repair genetic defects in these attenuated *L. donovani* isolates to identify key visceral disease–associated genetic determinants.

One of the challenges of studying the genome of *Leishmania* is that the majority of the genes do not have homology with genes of known function from higher eukaryotes. Indeed, although several species-specific genes are associated with visceral disease in animal models [10], [81], [82], the role of the majority of them remains to be determined. Gene function determination is required, potentially aided by the identification of relevant protein-protein interactions.

Visceral leishmaniasis is one of the most lethal neglected tropical diseases [3] and is closely associated with poverty [99]. A better understanding of the factors that mediate visceral disease will help guide the identification of better drug targets, immunomodulators, and epidemiologic markers for virulence and potential vaccines, which will support disease elimination efforts that would have a significant impact in the poorest regions of endemic countries.

## References

[1] Murray HW, Berman JD, Davies CR, Saravia NG (2005) Advances in leishmaniasis. Lancet 366: 1561–1577. 16257344 10.1016/S0140-6736(05)67629-5

[2] Alvar J, Vélez ID, Bern C, Herrero M, Desjeux P, et al (2012) Leishmaniasis worldwide and global estimates of its incidence. PLoS ONE 7: e35671 doi:10.1371/journal.pone.0035671. 22693548 10.1371/journal.pone.0035671 PMC3365071

[3] Mathers CD, Ezzati M, Lopez AD (2007) Measuring the burden of neglected tropical diseases: the global burden of disease framework. PLoS Negl Trop Dis 1: e114 doi:10.1371/journal.pntd.0000114. 18060077 10.1371/journal.pntd.0000114 PMC2100367

[4] Bern C, Maguire JH, Alvar J (2008) Complexities of assessing the disease burden attributable to leishmaniasis. PLoS Negl Trop Dis 2: e313 doi:10.1371/journal.pntd.0000313. 18958165 10.1371/journal.pntd.0000313 PMC2569207

[5] Bates PA (2007) Transmission of Leishmania metacyclic promastigotes by phlebotomine sand flies. Int J Parasitol 37: 1097–1106. 17517415 10.1016/j.ijpara.2007.04.003 PMC2675784

[6] de Souza EP, Pereira APE, Machado FCS, Melo MF, Souto-Padron T, et al (2001) Occurrence of Leishmania donovani parasitemia in plasma of infected hamsters. Acta Trop 80: 69–75. 11495646 10.1016/s0001-706x(01)00150-4

[7] Thalhofer CJ, Chen YN, Sudan B, Love-Homan L, Wilson ME (2011) Leukocytes infiltrate the skin and draining lymph nodes in response to the protozoan Leishmania infantum chagasi. Infect Immun 79: 108–117. 20937764 10.1128/IAI.00338-10 PMC3019875

[8] Peters NC, Kimblin N, Secundino N, Kamhawi S, Lawyer P, et al (2009) Vector transmission of Leishmania abrogates vaccine-induced protective immunity. PLoS Pathog 5: e1000484 doi:10.1371/journal.ppat.1000484. 19543375 10.1371/journal.ppat.1000484 PMC2691580

[9] Ribeiro-Gomes FL, Peters NC, Debrabant A, Sacks DL (2012) Efficient capture of infected neutrophils by dendritic cells in the skin inhibits the early anti-Leishmania response. PLoS Pathog 8: e1002536 doi:10.1371/journal.ppat.1002536. 22359507 10.1371/journal.ppat.1002536 PMC3280984

[10] Zhang WW, Mendez S, Ghosh A, Myler P, Ivens A, et al (2003) Comparison of the A2 gene locus in Leishmania donovani and Leishmania major and its control over cutaneous infection. J Biol Chem 278: 35508–35515. 12829719 10.1074/jbc.M305030200

[11] Laskay T, Diefenbach A, Rollinghoff M, Solbach W (1995) Early parasite containment is decisive for resistance to Leishmania major infection. Eur J Immunol 25: 2220–2227. 7664785 10.1002/eji.1830250816

[12] Melby PC, Yang YZ, Cheng J, Zhao WG (1998) Regional differences in the cellular immune response to experimental cutaneous or visceral infection with Leishmania donovani. Infect Immun 66: 18–27. 9423834 10.1128/iai.66.1.18-27.1998 PMC107853

[13] Ahmed S, Colmenares M, Soong L, Goldsmith-Pestana K, Munstermann L, et al (2003) Intradermal infection model for pathogenesis and vaccine studies of murine visceral leishmaniasis. Infect Immun 71: 401–410. 12496190 10.1128/IAI.71.1.401-410.2003 PMC143149

[14] Gomes R, Teixeira C, Teixeira MJ, Oliveira F, Menezes MJ, et al (2008) Immunity to a salivary protein of a sand fly vector protects against the fatal outcome of visceral leishmaniasis in a hamster model. Proc Natl Acad Sci U S A 105: 7845–7850. 18509051 10.1073/pnas.0712153105 PMC2397325

[15] Maia C, Seblova V, Sadlova J, Votypka J, Volf P (2011) Experimental transmission of Leishmania infantum by two major vectors: a comparison between a viscerotropic and a dermotropic strain. PLoS Negl Trop Dis 5: e1181 doi:10.1371/journal.pntd.0001181. 21695108 10.1371/journal.pntd.0001181 PMC3114756

[16] Engwerda CR, Ato M, Kaye PM (2004) Macrophages, pathology and parasite persistence in experimental visceral leishmaniasis. Trends Parasitol 20: 524–530. 15471704 10.1016/j.pt.2004.08.009

[17] Nieto A, Dominguez-Bernal G, Orden JA, De La Fuente R, Madrid-Elena N, et al (2011) Mechanisms of resistance and susceptibility to experimental visceral leishmaniosis: BALB/c mouse versus syrian hamster model. Vet Res 42: 39. 21345200 10.1186/1297-9716-42-39 PMC3052183

[18] Zhang WW, Matlashewski G (2001) Characterization of the A2-A2rel gene cluster in Leishmania donovani: involvement of A2 in visceralization during infection. Mol Microbiol 39: 935–948. 11251814 10.1046/j.1365-2958.2001.02286.x

[19] Scott P (1985) Impaired macrophage leishmanicidal activity at cutaneous temperature. Parasite Immunol 7: 277–288. 4011301 10.1111/j.1365-3024.1985.tb00076.x

[20] Wittner M, Tanowitz HB (2000) Leishmaniasis in infants and children. Semin Pediatr Infect Dis 11: 196–201.

[21] McCall LI, Matlashewski G (2010) Localization and induction of the A2 virulence factor in Leishmania: evidence that A2 is a stress response protein. Mol Microbiol 77: 518–530. 20497497 10.1111/j.1365-2958.2010.07229.x

[22] Callahan HL, Portal IF, Bensinger SJ, Grogl M (1996) Leishmania spp: temperature sensitivity of promastigotes in vitro as a model for tropism in vivo. Exp Parasitol 84: 400–409. 8948329 10.1006/expr.1996.0128

[23] Hoyer C, Mellenthin K, Schilhabel M, Platzer M, Clos J (2001) Use of genetic complementation to identify gene(s) which specify species-specific organ tropism of Leishmania. Med Microbiol Immunol 190: 43–46. 11770108 10.1007/s004300100077

[24] Beachy SH, Repasky EA (2011) Toward establishment of temperature thresholds for immunological impact of heat exposure in humans. Int J Hyperthermia 27: 344–352. 21591898 10.3109/02656736.2011.562873 PMC3620730

[25] Sarkar A, Ghosh S, Pakrashi S, Roy D, Sen S, et al (2012) Leishmania strains causing self-healing cutaneous leishmaniasis have greater susceptibility towards oxidative stress. Free Radic Res 46: 665–673. 22385212 10.3109/10715762.2012.668186

[26] De Trez C, Magez S, Akira S, Ryffel B, Carlier Y, et al (2009) iNOS-producing inflammatory dendritic cells constitute the major infected cell type during the chronic Leishmania major infection phase of C57BL/6 resistant mice. PLoS Pathog 5: e1000494 doi:10.1371/journal.ppat.1000494. 19557162 10.1371/journal.ppat.1000494 PMC2695779

[27] Kaye P, Scott P (2011) Leishmaniasis: complexity at the host-pathogen interface. Nat Rev Microbiol 9: 604–615. 21747391 10.1038/nrmicro2608

[28] Taylor PR, Martinez-Pomares L, Stacey M, Lin HH, Brown GD, et al (2005) Macrophage receptors and immune recognition. Annu Rev Immunol 23: 901–944. 15771589 10.1146/annurev.immunol.23.021704.115816

[29] Olivier M, Tanner CE (1987) Susceptibilities of macrophage populations to infection in vitro by Leishmania donovani. Infect Immun 55: 467–471. 3804446 10.1128/iai.55.2.467-471.1987 PMC260352

[30] Redmond HP, Shou J, Gallagher HJ, Kelly CJ, Daly JM (1993) Macrophage-dependent candidacidal mechanisms in the murine system. Comparison of murine Kupffer cell and peritoneal macrophage candidacidal mechanisms. J Immunol 150: 3427–3433. 8385685

[31] Liu GW, Xia XP, Gong SL, Zhao Y (2006) The macrophage heterogeneity: difference between mouse peritoneal exudate and splenic F4/80(+) macrophages. J Cell Physiol 209: 341–352. 16883572 10.1002/jcp.20732

[32] Rutherford MS, Witsell A, Schook LB (1993) Mechanisms generating functionally heterogeneous macrophages: chaos revisited. J Leukoc Biol 53: 602–618. 8501399 10.1002/jlb.53.5.602

[33] Badaró R, Jones TC, Lorenço R, Cerf BJ, Sampaio D, et al (1986) A prospective study of visceral leishmaniasis in an endemic area of Brazil. J Infect Dis 154: 639–649. 3745974 10.1093/infdis/154.4.639

[34] Jeronimo SMB, Duggal P, Ettinger NA, Nascimento ET, Monteiro GR, et al (2007) Genetic predisposition to self-curing infection with the protozoan Leishmania chagasi: a genomewide scan. J InfectDis 196: 1261–1269. 10.1086/521682 PMC2330094 17955446

[35] Jamieson SE, Miller EN, Peacock CS, Fakiola M, Wilson ME, et al (2007) Genome-wide scan for visceral leishmaniasis susceptibility genes in Brazil. Genes Immun 8: 84–90. 17122780 10.1038/sj.gene.6364357 PMC2495017

[36] Sakthianandeswaren A, Foote SJ, Handman E (2009) The role of host genetics in leishmaniasis. Trends Parasitol 25: 383–391. 19617002 10.1016/j.pt.2009.05.004

[37] Blackwell JM, Fakiola M, Ibrahim ME, Jamieson SE, Jeronimo SB, et al (2009) Genetics and visceral leishmaniasis: of mice and man. Parasite Immunol 31: 254–266. 19388946 10.1111/j.1365-3024.2009.01102.x PMC3160815

[38] Lipoldova M, Demant P (2006) Genetic susceptibility to infectious disease: lessons from mouse models of leishmaniasis. Nat Rev Genet 7: 294–305. 16543933 10.1038/nrg1832

[39] Bucheton B, Abel L, Kheir MM, Mirgani A, El-Safi SH, et al (2003) Genetic control of visceral leishmaniasis in a Sudanese population: candidate gene testing indicates a linkage to the NRAMP1 region. Genes Immun 4: 104–109. 12618857 10.1038/sj.gene.6363927

[40] Karplus TM, Jeronimo SMB, Chang H, Helms BK, Burns TL, et al (2002) Association between the tumor necrosis factor locus and the clinical outcome of Leishmania chagasi infection. Infect Immun 70: 6919–6925. 12438370 10.1128/IAI.70.12.6919-6925.2002 PMC133071

[41] Mohamed HS, Ibrahim ME, Miller EN, Peacock CS, Khalil EAG, et al (2003) Genetic susceptibility to visceral leishmaniasis in The Sudan: linkage and association with IL4 and IFNGR1. Genes Immun 4: 351–355. 12847550 10.1038/sj.gene.6363977

[42] Frade AF, de Oliveira LC, Costa DL, Costa CHN, Aquino D, et al (2011) TGFB1 and IL8 gene polymorphisms and susceptibility to visceral leishmaniasis. Infect Genet Evol 11: 912–916. 21376140 10.1016/j.meegid.2011.02.014

[43] Bucheton B, Argiro L, Chevillard C, Marquet S, Kheir MM, et al (2007) Identification of a novel G245R polymorphism in the IL-2 receptor beta membrane proximal domain associated with human visceral leishmaniasis. Genes Immun 8: 79–83. 17108990 10.1038/sj.gene.6364355

[44] Mehrotra S, Fakiola M, Oommen J, Jamieson SE, Mishra A, et al (2011) Genetic and functional evaluation of the role of CXCR1 and CXCR2 in susceptibility to visceral leishmaniasis in north-east India. BMC Med Genet 12: 162. 22171941 10.1186/1471-2350-12-162 PMC3260103

[45] Alonso DP, Ferreira AFB, Ribolla PEM, Santos IKFDM, Cruz MDSPE, et al (2007) Genotypes of the mannan-binding lectin gene and susceptibility to visceral leishmaniasis and clinical complications. J Infect Dis 195: 1212–1217. 17357060 10.1086/512683

[46] Mehrotra S, Fakiola M, Mishra A, Sudarshan M, Tiwary P, et al (2012) Genetic and functional evaluation of the role of DLL1 in susceptibility to visceral leishmaniasis in India. Infect Genet Evol 12: 1195–1201. 22561395 10.1016/j.meegid.2012.04.017 PMC3651914

[47] Desjeux P, Alvar J (2003) Leishmania/HIV co-infections: epidemiology in Europe. Ann Trop Med Parasitol 97 Suppl 1: 3–15. 14678629 10.1179/000349803225002499

[48] Osorio Y, Rodriguez LD, Bonilla DL, Peniche AG, Henao H, et al (2012) Congenital transmission of experimental leishmaniasis in a hamster model. Am J Trop Med Hyg 86: 812–820. 22556079 10.4269/ajtmh.2012.11-0458 PMC3335685

[49] Dye C, Williams BG (1993) Malnutrition, age and the risk of parasitic disease: visceral leishmaniasis revisited. Proc Biol Sci 254: 33–39. 8265673 10.1098/rspb.1993.0123

[50] Maciel BLL, Lacerda HG, Queiroz JW, Galvao J, Pontes NN, et al (2008) Association of nutritional status with the response to infection with Leishmania chagasi. Am J Trop Med Hyg 79: 591–598. 18840750

[51] Müller I, Hailu A, Choi B-S, Abebe T, Fuentes JM, et al (2008) Age-related alteration of arginase activity impacts on severity of leishmaniasis. PLoS Negl Trop Dis 2: e235 doi:10.1371/journal.pntd.0000235. 18478052 10.1371/journal.pntd.0000235 PMC2359854

[52] Singh N, Samant M, Gupta SK, Kumar A, Dube A (2007) Age-influenced population kinetics and immunological responses of Leishmania donovani in hamsters. Parasitol Res 101: 919–924. 17484071 10.1007/s00436-007-0562-3

[53] Gramiccia M (2003) The identification and variability of the parasites causing leishmaniasis in HIV-positive patients in Italy. Ann Trop Med Parasitol 97: 65–73. 14678634 10.1179/000349803225002543

[54] Santos-Oliveira JR, Da-Cruz AM, Pires LHS, Cupolillo E, Kuhls K, et al (2011) Case report: atypical lesions as a sign of cutaneous dissemination of visceral leishmaniasis in a human immunodeficiency virus-positive patient simultaneously infected by two viscerotropic Leishmania species. Am J Trop Med Hyg 85: 55–59. 21734124 10.4269/ajtmh.2011.10-0398 PMC3122343

[55] Stanley AC, Engwerda CR (2007) Balancing immunity and pathology in visceral leishmaniasis. Immunol Cell Biol 85: 138–147. 17146466 10.1038/sj.icb7100011

[56] Diefenbach A, Schindler H, Rollinghoff M, Yokoyama WM, Bogdan C (1999) Requirement for type 2 NO synthase for IL-12 signaling in innate immunity. Science 284: 951–955. 10320373 10.1126/science.284.5416.951

[57] Singh OP, Gidwani K, Kumar R, Nylen S, Jones SL, et al (2012) Reassessment of immune correlates in human visceral leishmaniasis as defined by cytokine release in whole blood. Clin Vaccine Immunol 19: 961–966. 22539471 10.1128/CVI.00143-12 PMC3370446

[58] Verma S, Kumar R, Katara GK, Singh LC, Negi NS, et al (2010) Quantification of parasite load in clinical samples of leishmaniasis patients: IL-10 level correlates with parasite load in visceral leishmaniasis. PLoS ONE 5: e10107 doi:10.1371/journal.pone.0010107. 20404924 10.1371/journal.pone.0010107 PMC2852412

[59] Nylen S, Sacks D (2007) Interleukin-10 and the pathogenesis of human visceral leishmaniasis. Trends Immunol 28: 378–384. 17689290 10.1016/j.it.2007.07.004

[60] Warburg A, Saraiva E, Lanzaro GC, Titus RG, Neva F (1994) Saliva of Lutzomyia longipalpis sibling species differs in its composition and capacity to enhance leishmaniasis. Philos Trans R Soc Lond B Biol Sci 345: 223–230. 7972360 10.1098/rstb.1994.0097

[61] Rohoušová I, Subrahmanyam S, Volfová V, Mu J, Volf P, et al (2012) Salivary gland transcriptomes and proteomes of Phlebotomus tobbi and Phlebotomus sergenti, vectors of leishmaniasis. PLoS Negl Trop Dis 6: e1660 doi:10.1371/journal.pntd.0001660. 22629480 10.1371/journal.pntd.0001660 PMC3358328

[62] Vinhas V, Andrade BB, Paes F, Bomura A, Clarencio J, et al (2007) Human anti-saliva immune response following experimental exposure to the visceral leishmaniasis vector, Lutzomyia longipalpis. Eur J Immunol 37: 3111–3121. 17935072 10.1002/eji.200737431

[63] Gomes RB, Brodskyn U, de Oliveira CI, Costa J, Miranda JC, et al (2002) Seroconversion against Lutzomyia longipalpis saliva concurrent with the development of anti-Leishmania chagasi delayed-type hypersensitivity. J Infect Dise 186: 1530–1534. 10.1086/344733 12404176

[64] Aquino DMC, Caldas AJM, Miranda JC, Silva AAM, Barral-Netto M, et al (2010) Short report: epidemiological study of the association between anti-Lutzomyia longipalpis saliva antibodies and development of delayed-type hypersensitivity to Leishmania antigen. Am J Trop Med Hyg 83: 825–827. 20889873 10.4269/ajtmh.2010.10-0182 PMC2946750

[65] Gradoni L, Gramiccia M (1994) Leishmania infantum tropism: strain genotype or host immune status? Parasitol Today 10: 264–267. 10.1016/0169-4758(94)90142-2 15275441

[66] Sulahian A, Garin YJF, Pratlong F, Dedet JP, Derouin F (1997) Experimental pathogenicity of viscerotropic and dermotropic isolates of Leishmania infantum from immunocompromised and immunocompetent patients in a murine model. FEMS Immunol Med Microbiol 17: 131–138. 9093833 10.1111/j.1574-695X.1997.tb01005.x

[67] Charest H, Matlashewski G (1994) Developmental gene expression in Leishmania donovani: differential cloning and analysis of an amastigote-stage-specific gene. Mol Cell Biol 14: 2975–2984. 7545921 10.1128/mcb.14.5.2975-2984.1994 PMC358665

[68] Ghedin E, Zhang WW, Charest H, Sundar S, Kenney RT, et al (1997) Antibody response against a Leishmania donovani amastigote-stage-specific protein in patients with visceral leishmaniasis. Clin Diagn Lab Immunol 4: 530–535. 9302200 10.1128/cdli.4.5.530-535.1997 PMC170587

[69] Charest H, Zhang WW, Matlashewski G (1996) The developmental expression of Leishmania donovani A2 amastigote-specific genes is post-transcriptionally mediated and involves elements located in the 3′-untranslated region. J Biol Chem 271: 17081–17090. 8663340 10.1074/jbc.271.29.17081

[70] Zhang WW, Charest H, Ghedin E, Matlashewski G (1996) Identification and overexpression of the A2 amastigote-specific protein in Leishmania donovani. Mol Biochem Parasitol 78: 79–90. 8813679 10.1016/s0166-6851(96)02612-6

[71] Zhang WW, Matlashewski G (1997) Loss of virulence in Leishmania donovani deficient in an amastigote-specific protein, A2. Proc Natl Acad Sci U S A 94: 8807–8811. 9238059 10.1073/pnas.94.16.8807 PMC23140

[72] Mizbani A, Taslimi Y, Zahedifard F, Taheri T, Rafati S (2011) Effect of A2 gene on infectivity of the nonpathogenic parasite Leishmania tarentolae. Parasitol Res 109: 793–799. 21442256 10.1007/s00436-011-2325-4

[73] Sharma P, Gurumurthy S, Duncan R, Nakhasi HL, Salotra P (2010) Comparative in vivo expression of amastigote up regulated Leishmania genes in three different forms of Leishmaniasis. Parasitol Int 59: 262–4. 19963076 10.1016/j.parint.2009.11.003

[74] Charest H, Matlashewski G (1994) Developmental gene expression in Leishmania donovani: differential cloning and analysis of an amastigote-stage-specific gene. Mol Cell Biol 14: 2975–2984. 7545921 10.1128/mcb.14.5.2975-2984.1994 PMC358665

[75] Gosline SJC, Nascimento M, McCall LI, Zilberstein D, Thomas DY, et al (2011) Intracellular eukaryotic parasites have a distinct unfolded protein response. PLoS ONE 6: e19118 doi:10.1371/journal.pone.0019118. 21559456 10.1371/journal.pone.0019118 PMC3084755

[76] Barak E, Amin-Spector S, Gerliak E, Goyard S, Holland N, et al (2005) Differentiation of Leishmania donovani in host-free system: analysis of signal perception and response. Mol Biochem Parasitol 141: 99–108. 15811531 10.1016/j.molbiopara.2005.02.004

[77] McCall LI, Matlashewski G (2012) Involvement of the Leishmania donovani virulence factor A2 in protection against heat and oxidative stress. Exp Parasitol 132: 109–15. 22691540 10.1016/j.exppara.2012.06.001

[78] Fernandes AP, Coelho EA, Machado-Coelho GL, Grimaldi GJ, Gazzinelli RT (2012) Making an anti-amastigote vaccine for visceral leishmaniasis: rational, update and perspectives. Curr Opin Microbiol 15: 476–485. 22698479 10.1016/j.mib.2012.05.002

[79] Rogers MB, Hilley JD, Dickens NJ, Wilkes J, Bates PA, et al (2011) Chromosome and gene copy number variation allow major structural change between species and strains of Leishmania. Genome Res 21: 2129–2142. 22038252 10.1101/gr.122945.111 PMC3227102

[80] Peacock CS, Seeger K, Harris D, Murphy L, Ruiz JC, et al (2007) Comparative genomic analysis of three Leishmania species that cause diverse human disease. Nat Genet 39: 839–847. 17572675 10.1038/ng2053 PMC2592530

[81] Zhang WW, Peacock CS, Matlashewski G (2008) A genomic-based approach combining in vivo selection in mice to identify a novel virulence gene in Leishmania. PLoS Negl Trop Dis 2: e248 doi:10.1371/journal.pntd.0000248. 18545684 10.1371/journal.pntd.0000248 PMC2398785

[82] Zhang WW, Matlashewski G (2010) Screening Leishmania donovani-specific genes required for visceral infection. Mol Microbiol 77: 505–517. 20545850 10.1111/j.1365-2958.2010.07230.x

[83] Zhang WW, Chan KF, Song ZW, Matlashewski G (2011) Expression of a Leishmania donovani nucleotide sugar transporter in Leishmania major enhances survival in visceral organs. Exp Parasitol 129: 337–345. 21978449 10.1016/j.exppara.2011.09.010

[84] Downing T, Imamura H, Decuypere S, Clark TG, Coombs GH, et al (2011) Whole genome sequencing of multiple Leishmania donovani clinical isolates provides insights into population structure and mechanisms of drug resistance. Genome Res 21: 2143–2156. 22038251 10.1101/gr.123430.111 PMC3227103

[85] Rochette A, Raymond F, Ubeda JM, Smith M, Messier N, et al (2008) Genome-wide gene expression profiling analysis of Leishmania major and Leishmania infantum developmental stages reveals substantial differences between the two species. BMC Genomics 9: 255. 18510761 10.1186/1471-2164-9-255 PMC2453527

[86] Depledge DP, Evans KJ, Ivens AC, Aziz N, Maroof A, et al (2009) Comparative expression profiling of Leishmania: modulation in gene expression between species and in different host genetic backgrounds. PLoS Negl Trop Dis 3: e476 doi:10.1371/journal.pntd.0000476. 19582145 10.1371/journal.pntd.0000476 PMC2701600

[87] Lahav T, Sivam D, Volpin H, Ronen M, Tsigankov P, et al (2011) Multiple levels of gene regulation mediate differentiation of the intracellular pathogen Leishmania. FASEB J 25: 515–525. 20952481 10.1096/fj.10-157529 PMC6188352

[88] Drummelsmith J, Brochu V, Girard I, Messier N, Ouellette M (2003) Proteome mapping of the protozoan parasite Leishmania and application to the study of drug targets and resistance mechanisms. Mol Cell Proteomics 2: 146–155. 12644573 10.1074/mcp.M200085-MCP200

[89] Salotra P, Ralhan R, Sreenivas G (2000) Heat-stress induced modulation of protein phosphorylation in virulent promastigotes of Leishmania donovani. Int J Biochem Cell Biol 32: 309–316. 10716628 10.1016/s1357-2725(99)00134-x

[90] Gregory DJ, Sladek R, Olivier M, Matlashewski G (2008) Comparison of the effects of Leishmania major or Leishmania donovani infection on macrophage gene expression. Infect Immun 76: 1186–1192. 18086813 10.1128/IAI.01320-07 PMC2258831

[91] Anstead GM, Chandrasekar B, Zhao WG, Yang J, Perez LE, et al (2001) Malnutrition alters the innate immune response and increases early visceralization following Leishmania donovani infection. Infect Immun 69: 4709–4718. 11447142 10.1128/IAI.69.8.4709-4718.2001 PMC98556

[92] Kropf P, Fuentes JM, Fahnrich E, Arpa L, Herath S, et al (2005) Arginase and polyamine synthesis are key factors in the regulation of experimental leishmaniasis in vivo. FASEB J 19: 1000–1002. 15811879 10.1096/fj.04-3416fje

[93] Matte C, Olivier M (2002) Leishmania-induced cellular recruitment during the early inflammatory response: modulation of proinflammatory mediators. J Infect Dis 185: 673–681. 11865425 10.1086/339260

[94] McDowell MA, Marovich M, Lira R, Braun M, Sacks D (2002) Leishmania priming of human dendritic cells for CD40 ligand-induced interleukin-12p70 secretion is strain and species dependent. Infect Immun 70: 3994–4001. 12117904 10.1128/IAI.70.8.3994-4001.2002 PMC128119

[95] Meddeb-Garnaoui A, Zrelli H, Dellagi K (2009) Effects of tropism and virulence of Leishmania parasites on cytokine production by infected human monocytes. Clin Exp Immunol 155: 199–206. 19040614 10.1111/j.1365-2249.2008.03821.x PMC2675250

[96] Sadlova J, Yeo M, Seblova V, Lewis MD, Mauricio I, et al (2011) Visualisation of Leishmania donovani fluorescent hybrids during early stage development in the sand fly vector. PLoS ONE 6: e19851 doi:10.1371/journal.pone.0019851. 21637755 10.1371/journal.pone.0019851 PMC3103508

[97] Akopyants NS, Kimblin N, Secundino N, Patrick R, Peters N, et al (2009) Demonstration of genetic exchange during cyclical development of Leishmania in the sand fly vector. Science 324: 265–268. 19359589 10.1126/science.1169464 PMC2729066

[98] Siriwardana HVYD, Thalagala N, Karunaweera ND (2010) Clinical and epidemiological studies on the cutaneous leishmaniasis caused by Leishmania (Leishmania) donovani in Sri Lanka. Ann Trop Med Parasitol 104: 213–223. 20507695 10.1179/136485910X12647085215615

[99] Alvar J, Yactayo S, Bern C (2006) Leishmaniasis and poverty. Trends Parasitol 22: 552–557. 17023215 10.1016/j.pt.2006.09.004

